# Case Report: Recurrent ventricular fibrillation induced by multivessel coronary artery spasm: a case supporting ICD for secondary prevention

**DOI:** 10.3389/fphys.2026.1808973

**Published:** 2026-06-12

**Authors:** Ling Li, Xiaoyan Chen, Yue Deng

**Affiliations:** 1 Laboratory Department, Tanzhou People’s Hospital of Zhongshan, Zhongshan City Hospital of Integration of TCM & Western Medicine, Zhongshan, Guangdong, China; 2 Department of Cardiology, Ningyuan County People’s Hospital, Yongzhou, Hunan, China; 3 Department of Cardiology, Baiyun Branch of the Third Affiliated Hospital of Guangzhou Medical University (Baiyun Maternal and Child Health Hospital, Guangzhou, Guangdong, China

**Keywords:** coronary artery spasm, implantable cardioverter-defibrillator, sudden cardiac death, vasospastic angina, ventricular fibrillation

## Abstract

**Background:**

Coronary artery spasm (CAS), also known as vasospastic angina, is a functional coronary disorder that can precipitate transient myocardial ischemia and life-threatening ventricular arrhythmias. Although generally reversible, severe or multivessel CAS may lead to sudden cardiac death, and its optimal management remains controversial.

**Case presentation:**

We report a case of a 71-year-old man of Chinese ethnicity with a history of atrial fibrillation ablation and chronic obstructive pulmonary disease who presented with recurrent episodes of chest discomfort followed by sudden cardiac arrest. The patient experienced multiple episodes of ventricular tachycardia and ventricular fibrillation requiring repeated defibrillation and advanced cardiopulmonary resuscitation. Initial coronary angiography showed no significant fixed coronary stenosis. However, during recurrent ischemic episodes accompanied by dynamic ST-segment elevation, emergent repeat angiography demonstrated severe multivessel coronary spasm, including near-total occlusion of the mid left anterior descending artery and complete occlusion of the distal right coronary artery. Intracoronary nitrate administration promptly relieved the spasm and restored coronary flow. Despite intensive medical therapy with calcium channel blockers and nitrates and withdrawal of potential triggering agents, the patient suffered recurrent malignant ventricular arrhythmias. He ultimately underwent implantable cardioverter-defibrillator (ICD) implantation for secondary prevention. No ICD therapies were recorded during follow-up.

**Conclusion:**

This case highlights that multivessel coronary artery spasm can provoke recurrent malignant ventricular arrhythmias even in the absence of fixed coronary stenosis. In patients with CAS complicated by recurrent ventricular fibrillation despite optimal medical therapy, ICD implantation may be a reasonable strategy for secondary prevention of sudden cardiac death.

## Introduction

Coronary artery spasm (CAS), also known as vasospastic angina, is characterized by transient constriction of the epicardial coronary arteries, resulting in reversible myocardial ischemia (Lanza et al., 2011). Although often considered a functional disorder without fixed coronary stenosis, CAS can lead to severe clinical consequences, including malignant ventricular arrhythmias and sudden cardiac death (Matta et al., 2020).

The mechanisms of CAS are not fully elucidated but are thought to involve endothelial dysfunction, vascular smooth muscle hyperreactivity, and autonomic imbalance (de Silva et al., 2025). Multivessel coronary spasm represents a more diffuse pathological process and is associated with a higher risk of adverse cardiovascular events (Richardson et al., 2012). Transient but profound ischemia during spasm may create an unstable electrophysiological substrate, predisposing patients to ventricular tachycardia or ventricular fibrillation.

Diagnosis of CAS is challenging, as coronary angiography may appear normal outside of symptomatic episodes. Calcium channel blockers and nitrates are the mainstay of treatment; however, a small proportion of patients experience recurrent, life-threatening arrhythmias despite optimal medical therapy. The role of implantable cardioverter-defibrillators (ICDs) in this population remains uncertain.

Here, we present a case of multivessel CAS complicated by recurrent ventricular fibrillation, highlighting the malignant potential of this condition and the possible role of ICD implantation for secondary prevention.

## Case presentation

A 71-year-old man was admitted with a 5-day history of recurrent posterior neck discomfort accompanied by bilateral upper limb pain. His medical history included atrial fibrillation treated with radiofrequency ablation and chronic obstructive pulmonary disease managed with inhaled fluticasone–vilanterol. He was a current smoker. On admission, physical examination and baseline electrocardiography showed sinus rhythm without ischemic changes.

On day 1, the patient suddenly developed chest tightness followed by loss of consciousness and circulatory collapse. Cardiac monitoring revealed ventricular tachycardia, and successful resuscitation was achieved with cardiopulmonary resuscitation, endotracheal intubation, and electrical cardioversion. Post-resuscitation electrocardiography demonstrated sinus rhythm with frequent premature ventricular contractions. Cardiac biomarkers showed elevated cardiac troponin I (2.794 ng/mL), while transthoracic echocardiography revealed preserved left ventricular systolic function without regional wall motion abnormalities.

**Figure 1:**
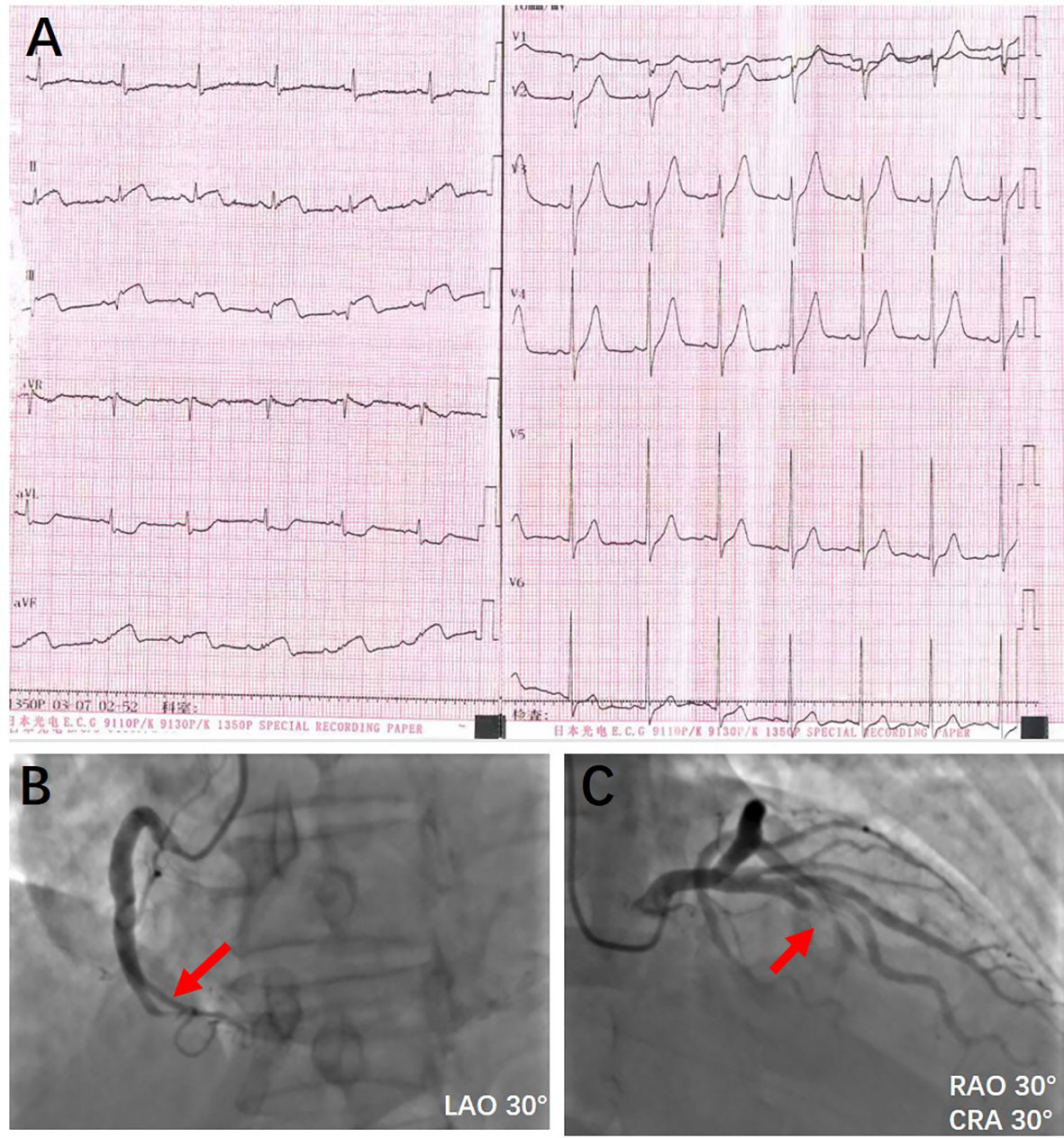
Admission electrocardiogram and initial coronary angiography. **(A)** Electrocardiogram obtained on admission showing sinus rhythm without ischemic changes. **(B, C)** Coronary angiography demonstrating no significant fixed stenosis in the left anterior descending artery, left circumflex artery, or right coronary artery.

During intensive care unit monitoring, the patient experienced an episode of atrial fibrillation with rapid ventricular response, which was successfully converted to sinus rhythm with amiodarone. Subsequent coronary angiography revealed no significant fixed coronary artery stenosis on day 3. However, recurrent episodes of chest pain were later accompanied by dynamic ST-segment elevation in the inferior leads on day 5. Emergency repeat angiography demonstrated severe multivessel coronary spasm, including near-total occlusion of the mid left anterior descending artery and complete occlusion of the distal right coronary artery (**Figure 1**). Intracoronary nitroglycerin (100 micrograms) administration promptly relieved the multivessel coronary spasm and restored coronary flow (**Figure 2**).

**kFigure 2:**
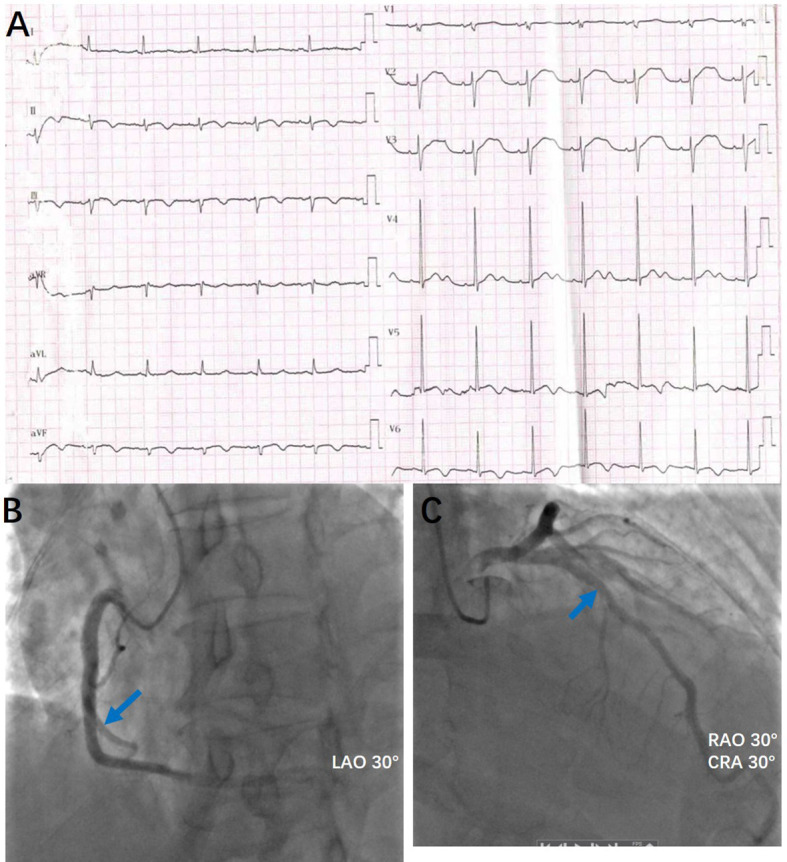
Changes in electrocardiography and coronary angiography after intracoronary nitroglycerin administration. **(A)** Electrocardiogram after intracoronary injection of nitroglycerin (200 μg) showing sinus rhythm with T-wave inversion in multiple leads. **(B)** Coronary angiography demonstrating resolution of distal right coronary artery occlusion after nitroglycerin administration (blue arrow). **(C)** Coronary angiography showing relief of mid–left anterior descending artery occlusion following nitroglycerin administration (blue arrow).

**Figure 3:**
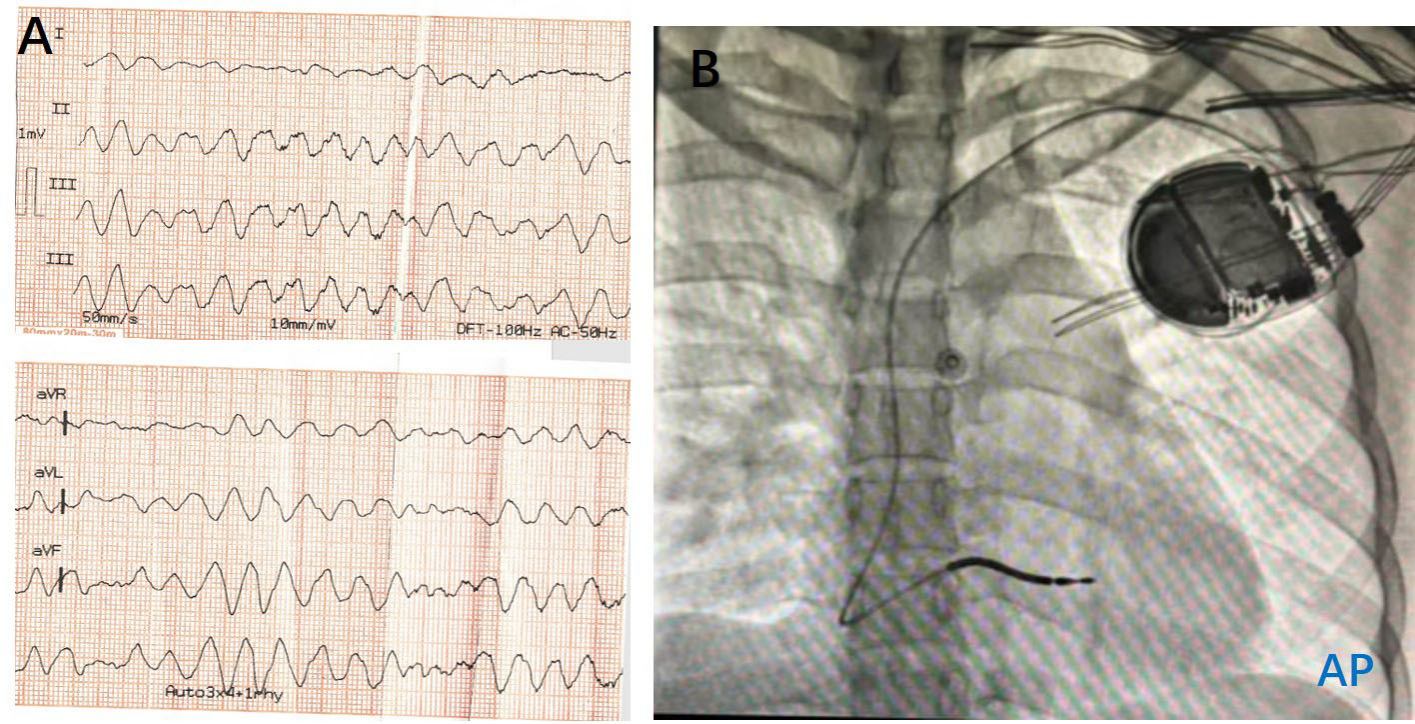
Ventricular fibrillation recorded on telemetry and implantable cardioverter-defibrillator (ICD) imaging. **(A)** Limb-lead electrocardiogram captured during an episode of ventricular fibrillation. **(B)** Post-procedural imaging showing the implanted ICD.

**Figure 4:**
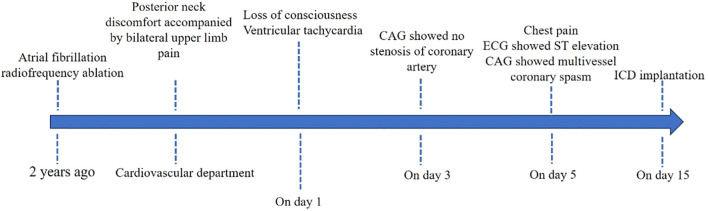
A timeline of this patient in diagnosis and management.

Despite intensive medical therapy with diltiazem (oral, 180 mg/day) and isosorbide mononitrate (oral, 50 mg/day) and discontinuation of potential triggering agents, the patient developed recurrent ventricular fibrillation with hemodynamic collapse, requiring repeated defibrillation and advanced life support (**Figure 3**). He was subsequently transferred to a tertiary center, where an implantable cardioverter-defibrillator was implanted for secondary prevention on day 15. **Figure 4** showed the timeline of this patient in diagnosis and management (**Figure 4**). The patient recovered uneventfully, and no ICD therapies were recorded during the follow-up.

## Discussion

This case illustrates the malignant potential of coronary artery spasm (CAS), particularly when multivessel involvement leads to recurrent ventricular fibrillation and aborted sudden cardiac death. Although CAS is traditionally regarded as a functional and reversible coronary disorder, this report underscores that it can serve as a potent trigger for life-threatening ventricular arrhythmias even in the absence of fixed coronary artery stenosis (Chevalier et al., 2002).

A major diagnostic challenge in this patient was the initial absence of significant coronary lesions on angiography. CAS is inherently dynamic, and coronary arteries may appear normal outside of active spasm episodes. In this context, transient ST-segment elevation during symptoms and the dramatic response to intracoronary nitrates were key diagnostic features. Multivessel spasm, as observed in this case, reflects diffuse coronary hyperreactivity and has been consistently associated with a higher risk of malignant arrhythmias and sudden cardiac death compared with single-vessel involvement. Regarding provocative testing and electrophysiological (EP) studies, current guidelines recognize intracoronary acetylcholine or ergonovine provocation as the diagnostic gold standard for coronary artery spasm (CAS); however, in patients presenting with aborted sudden cardiac death and spontaneous episodes of dynamic ST-segment elevation with angiographically confirmed multivessel spasm—as in our case—provocative testing is often deferred due to safety concerns and because the diagnosis is already established. Similarly, EP studies are not routinely mandated for risk stratification in CAS patients with documented ventricular fibrillation, as the arrhythmia typically arises from acute ischemia rather than a fixed re-entrant substrate. In our patient, given recurrent ventricular fibrillation despite optimal medical therapy, ICD implantation for secondary prevention was pursued without prior EP study.

The mechanisms by which CAS precipitates ventricular arrhythmias are likely multifactorial (Nishi et al., 2023). Acute transmural ischemia during spasm can cause electrical instability through dispersion of repolarization, intracellular calcium overload, and autonomic nervous system activation. Repeated ischemia–reperfusion episodes may further contribute to myocardial injury, local fibrosis, and the development of a persistent arrhythmogenic substrate, thereby lowering the threshold for ventricular fibrillation. In addition, autonomic imbalance and endothelial dysfunction may amplify both vasomotor instability and electrical vulnerability.

Calcium channel blockers and nitrates remain the cornerstone of therapy for CAS and are generally effective in preventing recurrent ischemic episodes (Maseri et al., 1978; Yamashina et al., 2013). However, a subset of patients exhibits refractory or recurrent spasm despite optimized medical therapy. In such high-risk individuals, particularly those with documented ventricular tachycardia or ventricular fibrillation, the role of ICDs remains controversial due to limited guideline-directed recommendations (Cheng, 1998). Nevertheless, accumulating evidence from case series and observational studies suggests that ICD implantation may provide effective secondary prevention in patients with CAS complicated by aborted sudden cardiac death.

Of note, while the ESC 2022 and AHA/ACC/HRS 2017 guidelines support ICD implantation for secondary prevention of SCD, they do not specifically address CAS (Zeppenfeld et al., 2022; Rao et al., 2025). However, the Japanese Circulation Society (JCS) guidelines for vasospastic angina have identified a high-risk subgroup of patients with CAS who may be vulnerable to SCD and may benefit from ICD implantation (Hokimoto et al., 2023). ICD implantation is generally considered for secondary prevention in survivors of SCD or sustained VT when the cause is not completely reversible or when optimal medical therapy fails to prevent recurrence. In the context of CAS, the decision is heavily individualized: if malignant arrhythmias occur despite maximal vasodilator therapy (calcium channel blockers and nitrates) and avoidance of triggers, guideline recommendations for secondary prevention in patients with structural heart disease are often extrapolated to this setting, supporting ICD implantation.

In our case, recurrent ventricular fibrillation occurred despite optimized medical therapy and withdrawal of potential triggers, and no reversible cause such as acute coronary syndrome or significant fixed coronary artery disease was identified. Therefore, ICD implantation was pursued as a reasonable strategy for secondary prevention, aligning with the principle that life-threatening arrhythmias in the absence of a definitively and sustainably correctable cause warrant device therapy.

### Limitations

This case has two main limitations. First, stress/rest cardiac magnetic resonance was not performed due to acute clinical instability and the patient’s non-MR-conditional ICD. Second, an electrophysiological study was not performed because the arrhythmia mechanism was clearly established by angiography showing active multivessel spasm, and a negative study would not have changed the decision for ICD implantation. Therefore, we cannot completely exclude concomitant structural or electrophysiological abnormalities.

## Conclusions

In conclusion, multivessel CAS should be recognized as a high-risk condition capable of inducing recurrent malignant ventricular arrhythmias. Early recognition, aggressive medical management, and individualized consideration of ICD implantation are crucial to improving outcomes in selected patients.

## Data Availability

The raw data supporting the conclusions of this article will be made available by the authors, without undue reservation.
